# Supplementary material for: Association between Geriatric Nutrition Risk Index and The Presence of Sarcopenia in People with Type 2 Diabetes Mellitus: A Cross-Sectional Study
Source: Nutrients. 2021 Oct 22;13(11):3729. doi: 10.3390/nu13113729 (PMC8618310; doi:10.3390/nu13113729)
Supplement: Supplementary file 1 [file nutrients-13-03729-s001.zip › GNRIsarcopenia S table (Nutrients).pdf]

Table S1. Clinical characteristics according to geriatric nutritional risk index separated by sex

	Men			Women		
	GNRI ≥98<br>N = 282	GNRI <98<br>N = 19	*p*	GNRI ≥98<br>N = 217	GNRI <98<br>N = 8	*p*
Age (years)	67.3 (10.9)	73.5 (8.4)	0.016	65.9 (10.9)	72.5 (7.0)	0.092
Duration of diabetes (years)	15.1 (9.7)	16.6 (11.9)	0.540	13.6 (10.7)	15.0 (11.1)	0.714
Family history of diabetes (-/+)	175/107	11/8	0.096	111/106	5/3	0.787
Height (cm)	167.2 (5.8)	162 (5.6)	0.002	153.3 (5.8)	156.0 (2.7)	0.200
Body weight (kg)	67.8 (11.2)	52.2 (6.8)	<0.001	59.3 (12.4)	45.0 (6.3)	0.001
Body mass index (kg/m^2)	24.2 (3.5)	19.7 (2.4)	<0.001	25.2 (5.0)	18.5 (2.6)	<0.001
SBP (mmHg)	131.8 (16.5)	136.3 (26.9)	0.281	134.8 (19.2)	127.5 (16.7)	0.292
DBP (mmHg)	77.0 (11.7)	71.7 (18.9)	0.070	76.7 (12.3)	69.0 (14.0)	0.086
Insulin (-/+)	217/65	11/8	0.110	165/52	4/4	0.209
SGLT2 inhibitor (-/+)	229/53	18/1	0.238	176/41	8/0	0.372
GLP-1 antagonist (-/+)	242/40	18/1	0.452	177/40	6/2	0.995
Antihypertensive drugs (-/+)	125/157	7/12	0.691	104/113	5/3	0.653
Presence of hypertension (-/+)	98/184	5/14	0.617	72/145	3/5	1.000
Smoking (-/+)	226/56	13/6	0.353	202/15	8/0	0.962
Habit of exercise (-/+)	139/143	11/8	0.625	118/99	8/0	0.029
HbA1c (mmol/mol)	56.6 (11.9)	63.9 (21.6)	0.016	56.5 (12.6)	72.5 (32.5)	0.001
HbA1c (%)	7.3 (1.1)	8.0 (2.0)	0.016	7.3 (1.1)	8.8 (3.0)	0.001

Plasma glucose (mmol/L)	8.4 (2.5)	9.9 (5.0)	0.023	8.0 (2.5)	9.9 (3.5)	0.034
Creatinine (umol/L)	83.9 (32.8)	104.8 (65.8)	0.014	59.2 (19.0)	56.9 (10.5)	0.734
eGFR (mL/min/1.73 m^2)	68.0 (19.6)	61.8 (28.3)	0.198	72.5 (18.8)	70.2 (13.4)	0.730
Uric acid (mmol/L)	328.7 (76.4)	306.8 (85.8)	0.230	288.9 (67.3)	228.3 (36.8)	0.012
Triglycerides (mmol/L)	1.6 (1.0)	1.2 (0.5)	0.080	1.4 (0.8)	1.4 (0.9)	0.892
HDL cholesterol (mmol/L)	1.5 (0.4)	1.7 (0.6)	0.047	1.6 (0.4)	1.5 (0.6)	0.307
C-reactive protein (ug/L)	1616.0 (3694.2)	15389.5 (31230.6)	<0.001	2747.6 (11063.3)	17537.5 (31528.0)	<0.001
Albumin (mg/L)	43.2 (3.0)	35.3 (4.0)	<0.001	42.6 (2.9)	35.8 (5.3)	<0.001
GNRI	116.1 (8.6)	94.7 (6.0)	<0.001	117.4 (10.4)	92.8 (5.0)	<0.001
Body fat mass (kg)	18.0 (7.3)	9.6 (5.4)	<0.001	21.5 (9.0)	11.2 (4.0)	0.001
Percent body fat mass (%)	25.9 (6.9)	17.7 (8.3)	<0.001	35.1 (7.7)	24.3 (6.4)	<0.001
Appendicular muscle mass (kg)	21.1 (3.1)	18.0 (2.5)	<0.001	15.0 (2.6)	12.7 (1.9)	0.013
SMI (kg/m^2)	7.5 (0.8)	6.8 (0.8)	<0.001	6.4 (0.9)	5.2 (0.7)	<0.001
Low skeletal muscle mass (-/+)	217/65	6/13	<0.001	172/45	3/5	0.018
Handgrip strength (kg)	33.6 (7.6)	26.7 (7.3)	<0.001	20.8 (5.1)	17.2 (4.8)	0.049
Low muscle strength (-/+)	223/59	8/11	<0.001	161/56	3/5	0.059
Presence of sarcopenia (-/+)	248/34	11/8	<0.001	196/21	4/4	0.003

Data was expressed as mean (standard deviation) or number. The difference between group was evaluated by Student's t-test or chi-square test. SBP, systolic blood pressure; DBP, diastolic blood pressure; eGFR, estimated glomerular filtration rate; HDL, high-density lipoprotein; GNRI, Geriatric Nutritional Risk Index; SMI, skeletal muscle mass index.

Table S2. Odds ratio of geriatric nutritional risk index on the presence of sarcopenia according to sex

Men	Model 1		Model 2		Model 3		Model 4	
	OR (95%CI)	*p*	OR (95%CI)	*p*	OR (95%CI)	*p*	OR (95%CI)	*p*
Low GNRI (<98)	5.30 (1.99-14.1)	<0.001	3.73 (1.25-11.1)	0.018	4.80 (1.52-15.1)	0.007	4.22 (1.26-14.1)	0.019
Age (years)	-	-	1.15 (1.10-1.21)	<0.001	1.14 (1.08-1.2)	<0.001	1.14 (1.08-1.20)	<0.001
Duration of diabetes (years)	-	-	-	-	1.03 (0.99-1.07)	0.184	1.03 (0.99-1.08)	0.107
Habit of exercise	-	-	-	-	1.77 (0.83-3.79)	0.140	1.75 (0.81-3.78)	0.153
Habit of smoking	-	-	-	-	0.29 (0.07-1.12)	0.072	0.29 (0.07-1.19)	0.086
HbA1c (mmol/mol)	-	-	-	-	-	-	1.03 (0.995-1.07)	0.090
Insulin treatment	-	-	-	-	-	-	0.61 (0.23-1.60)	0.317
Usage of SGLT2 inhibitor	-	-	-	-	-	-	0.52 (0.15-1.85)	0.314
Usage of GLP-1 antagonist	-	-	-	-	-	-	0.79 (0.19-3.20)	0.736

Men	Model 1		Model 2		Model 3		Model 4	
	OR (95%CI)	*p*	OR (95%CI)	*p*	OR (95%CI)	*p*	OR (95%CI)	*p*
GNRI	0.90 (0.86-0.95)	<0.001	0.91 (0.87-0.95)	<0.001	0.90 (0.96-0.95)	<0.001	0.90 (0.86-0.95)	<0.001
Age (years)	-	-	1.13 (1.07-1.19)	<0.001	1.10 (1.05-1.18)	<0.001	1.11 (1.05-1.17)	<0.001

Duration of diabetes (years)	-	-	-	-	1.02 (0.98-1.06)	0.286	1.03 (0.99-1.07)	0.163
Habit of exercise	-	-	-	-	1.51 (0.70-3.29)	0.297	0.15 (0.66-3.20)	0.352
Habit of smoking	-	-	-	-	0.21 (0.05-0.95)	0.043	0.18 (0.03-0.91)	0.038
HbA1c (mmol/mol)	-	-	-	-	-	-	1.03 (0.99-1.07)	0.150
Insulin treatment	-	-	-	-	-	-	0.54 (0.21-1.44)	0.218
Usage of SGLT2 inhibitor	-	-	-	-	-	-	0.75 (0.19-2.91)	0.676
Usage of GLP-1 antagonist	-	-	-	-	-	-	1.07 (0.26-4.52)	0.923

Women	Model 1		Model 2		Model 3		Model 4	
	OR (95%CI)	*p*	OR (95%CI)	*p*	OR (95%CI)	*p*	OR (95%CI)	*p*
Low GNRI (<98)	9.33 (2.17-40.1)	<0.001	7.00 (1.55-31.6)	0.011	8.70 (1.67-45.3)	0.010	11.3 (1.85-69.0)	0.009
Age (years)	-	-	1.09 (1.02-1.15)	0.006	1.08 (1.01-1.15)	0.032	1.08 (1.01-1.16)	0.035
Duration of diabetes (years)	-	-	-	-	1.06 (1.02-1.10)	0.002	1.05 (1.01-1.10)	0.009
Habit of exercise	-	-	-	-	0.72 (0.27-1.95)	0.521	0.82 (0.30-2.29)	0.706
Habit of smoking	-	-	-	-	4.46 (1.07-18.6)	0.040	5.26 (1.22-22.8)	0.026
HbA1c (mmol/mol)	-	-	-	-	-	-	0.98 (0.94-1.03)	0.472

Insulin treatment	-	-	-	-	-	-	1.19 (0.39-3.57)	0.762
Usage of SGLT2 inhibitor	-	-	-	-	-	-	0.59 (0.10-3.35)	0.553
Usage of GLP-1 antagonist	-	-	-	-	-	-	0.94 (0.21-4.17)	0.934

Women	Model 1		Model 2		Model 3		Model 4	
	OR (95%CI)	*p*	OR (95%CI)	*p*	OR (95%CI)	*p*	OR (95%CI)	*p*
GNRI	0.86 (0.81-0.92)	<0.001	0.87 (0.82-0.93)	<0.001	0.88 (0.83-0.94)	<0.001	0.86 (0.80-0.93)	<0.001
Age (years)	-	-	1.06 (0.99-1.13)	0.109	1.06 (0.98-1.13)	0.146	1.06 (0.98-1.14)	0.162
Duration of diabetes (years)	-	-	-	-	1.05 (1.01-1.09)	0.020	1.04 (1.00-1.09)	0.049
Habit of exercise	-	-	-	-	0.59 (0.21-1.65)	0.317	0.63 (0.22-1.83)	0.394
Habit of smoking	-	-	-	-	4.57 (0.98-21.4)	0.054	5.27 (1.03-27.0)	0.046
HbA1c (mmol/mol)	-	-	-	-	-	-	0.98 (0.93-1.02)	0.348
Insulin treatment	-	-	-	-	-	-	0.95 (0.30-3.03)	0.932
Usage of SGLT2 inhibitor	-	-	-	-	-	-	0.93 (0.14-6.05)	0.942
Usage of GLP-1 antagonist	-	-	-	-	-	-	1.05 (0.18-6.10)	0.958

Model 1 is unadjusted; Model 2 is adjusted for age, sex; Model 3 is adjusted for age, sex, duration of diabetes, habit of exercise, habit of

smoking; Model 4 is adjusted for age, sex, duration of diabetes, habit of exercise, habit of smoking, HbA1c, insulin treatment, usage of SGLT2 inhibitor, usage of GLP-1 antagonist. GNRI, Geriatric Nutritional Risk Index.
